# Serum Plasminogen Activator Inhibitor-1, α 1-Acid Glycoprotein, C-Reactive Protein, and Platelet Factor 4 Levels—Promising Molecules That Can Complete the “Puzzle” of the Biochemical Milieu in Severe Burns: Preliminary Results of a Cohort Prospective Study

**DOI:** 10.3390/jcm13102794

**Published:** 2024-05-09

**Authors:** Silviu Constantin Badoiu, Dan Mircea Enescu, Raluca Tatar, Iulia-Ioana Stanescu-Spinu, Daniela Miricescu, Maria Greabu, Ileana Paula Ionel, Viorel Jinga

**Affiliations:** 1 Department of Anatomy and Embriology, Faculty of Medicine, Carol Davila University of Medicine and Pharmacy, 8 Eroii Sanitari Blvd., 050474 Bucharest, Romania; silviu.badoiu@umfcd.ro; 2 Department of Plastic and Reconstructive Surgery, Life Memorial Hospital, 365 Grivitei Street, 010719 Bucharest, Romania; 3 Department of Plastic Reconstructive Surgery and Burns, Grigore Alexandrescu Clinical Emergency Hospital for Children, Faculty of Medicine, Carol Davila University of Medicine and Pharmacy, 37 Dionisie Lupu Street, 020021 Bucharest, Romania; dan.enescu@umfcd.ro (D.M.E.); raluca.tatar@umfcd.ro (R.T.); 4 Discipline of Physiology, Faculty of Dentistry, Carol Davila University of Medicine and Pharmacy, 8 Eroii Sanitari Blvd., 050474 Bucharest, Romania; 5 Discipline of Biochemistry, Faculty of Dentistry, Carol Davila University of Medicine and Pharmacy, 8 Eroii Sanitari Blvd., 050474 Bucharest, Romania; maria.greabu@umfcd.ro; 6 Discipline of General Nursing, Faculty of Midwifery and Nursing, Carol Davila University of Medicine and Pharmacy, 050474 Bucharest, Romania; ileana.ionel@umfcd.ro; 7 Department of Urology, Carol Davila University of Medicine and Pharmacy, 8 Eroii Sanitari Blvd., 050474 Bucharest, Romania; viorel.jinga@umfcd.ro; 8 Academy of Romanian Scientists, 3 Ilfov, 050085 Bucharest, Romania

**Keywords:** severe burns, inflammation, C-reactive protein (CRP), α 1-acid glycoprotein (AGP), plasminogen activator inhibitor-1 (PAI-1), platelet factor 4 (PF4)

## Abstract

**Background**: Burns represent a serious health problem, associated with multiple-organ failure, prolonged hospitalization, septic complications, and increased rate of mortality. The main aim of our study was to evaluate the levels of various circulating molecules in children with severe burns (more than 25% TBSA), in three different moments: 48 h, day 10, and day 21 post-burn. **Materials and Methods**: This study included 32 children with burns produced by flame, hot liquid, and electric arc and 21 controls. Serum plasminogen activator inhibitor-1 (PAI-1), α 1-acid glycoprotein (AGP), C-reactive protein (CRP), and platelet factor 4 (PF4) were detected using the Multiplex technique. Several parameters, such as fibrinogen, leucocyte count, thrombocyte count, triiodothyronine, thyroxine, and thyroid-stimulating hormone were also determined for each patient during hospitalization. **Results**: Significant statistical differences were obtained for CRP, AGP, and PF4 compared to the control group, in different moments of measurements. Negative correlations between CRP, AGP, and PF4 serum levels and burned body surface, and also the hospitalization period, were observed. **Discussions:** CRP levels increased in the first 10 days after burn trauma and then decreased after day 21. Serum PAI-1 levels were higher immediately after the burn and started decreasing only after day 10 post-burn. AGP had elevated levels 48 h after the burn, then decreased at 7–10 days afterwards, and once again increased levels after 21 days. PF4 serum levels increased after day 10 since the burning event. **Conclusions:** Serum CRP, AGP, PAI-1, and PF4 seem to be promising molecules in monitoring patients with a burn within the first 21 days.

## 1. Introduction

Burns represent one of the most severe forms of trauma [1]. Any severe burn ignites a systemic inflammatory response [2] and a hypermetabolic response [3] that have deleterious consequences on a patient’s biology [3], reflected in septic complications, multiple organ failure, prolonged hospitalization, and increased rate of death [4]. Survivors of major burn trauma exhibit physical and psychological permanent sequelae [5].

A better description of the evolution of patients with a burn from the very moment of the trauma can be made through an accurate biochemical and immunological characterization of the internal milieu.

The human body responds to trauma, tissue injury, surgical procedures, or malignant processes with the production of various acute-phase proteins including plasminogen activator inhibitor-1 (PAI-1), α 1-acid glycoprotein (AGP), C-reactive protein (CRP), and platelet factor 4 (PF4) [6]. Some of the acute-phase proteins have been used as diagnostic tools and prognostic markers in different types of trauma and infections [7]. The most commonly used is CRP, which has been considered a marker of the systemic inflammatory response and a prognostic factor in infections and burns for quite some time already [8,9]. Whenever a systemic inflammatory response appears (in trauma, extensive surgery, burns, sepsis, malignancies, etc.), T-cells (mainly in chronic situations) and macrophages (in acute situations) release several cytokines (such as IL-1, IL-2, IL-6, and TNF-α). Among other functions, these cytokines stimulate the release of CRP from hepatocytes. CRP attaches to the surface of microorganisms and altered cells, contributing to the activation of the complement system [10,11]. There has been a continuing interest in CRP’s value as a predictor for morbidity and mortality following severe burns [8,9,12]. The conclusions of previous studies were that (a) CRP did not accurately predict severe infection or sepsis in children with a severe burn [8]; (b) CRP accurately predicted mortality in severe burns [8,9]; (c) CRP had a better predictive potential in an elderly group with severe burns [9]; (d) CRP was correlated with the systemic inflammatory response [8,9,12]; (e) CRP was correlated with gender, race, age, and other factors [13]. PAI-1 is an acute-phase protein that inhibits fibrinolysis and has a procoagulant effect in severe trauma, severe burns, and sepsis [14]. In the systemic inflammatory response, some of the released cytokines (IL-6, TNF-α) stimulate PAI-1 expression in human adipose tissue, endothelial cells, activated macrophages, hepatocytes, etc. [14,15], promoting the procoagulant effect beyond the injured (burned) tissues [14].

In severe burns, as in other major trauma and just as in sepsis, there is a systemic coagulopathy that unveils. One can witness the activation of procoagulant mechanisms associated with the inhibition of natural fibrinolytic mechanisms [16]. It is one of the reasons why PAI-1 was considered to be associated with the injury severity and a possible predictor of morbidity and mortality in major trauma [17] and severe burns [18]. Besides that, PAI-1 was described as a good predictor of liver dysfunction in different circumstances: sepsis, hepatectomies, fatty liver disease, and hepatic fibrosis [19,20,21,22]. AGP is an acute-phase protein produced by hepatocytes, monocytes, and alveolar macrophages. AGP serum levels always increase in circumstances that induce a systemic inflammation: major trauma (including severe burns), malignancies, extensive surgery, and Crohn’s disease [23,24,25,26].

CRP and AGP were cited as biomarkers in the prediction of all-cause mortality [27,28]. Besides the fact that AGP levels increase in systemic inflammation, this molecule has anti-inflammatory and immunomodulatory effects [29,30,31], being a well-known inhibitor of neutrophiles and of the complement system [32,33]. AGP has another important function: it binds and transports endogen ligands related to inflammation and many drugs; thus, it is involved in the modulation of pharmacodynamics and pharmacokinetics of medicines [33].

PF4 is a small protein produced by activated platelets and dendritic cells [34,35,36]. PF4 levels increase in trauma and infections [37,38]. Being a cationic chemokine, PF4 binds to glycosaminoglycans (GAGs) on the surface of endothelial cells and on the surface of platelets. The result is the facilitation of platelet aggregation and edification of a thrombus. Hence, there is a powerful procoagulant effect [37,38]. The same cationic characteristic explains why PF4 binds to microorganisms (that have a negatively charged surface); this results in conformational changes in PF4 that is recognized as a foreign antigen by the immune system [38]. So, it is quite explicable why important amounts of PF4 are released from activated platelets, in infection. The same mechanism (binding to autoantibody-conformational changes in PF4 that is marked as non-self protein) is involved in autoimmune heparin-induced thrombocytopenia (HIT) and in vaccine-induced immune thrombotic thrombocytopenia (VITT) [38].

Although it was demonstrated that CRP, PAI-1, AGP, and PF4 were correlated with the inflammatory response, we considered their descriptive value and predictive value in burns to be underestimated. That is why we designed this pilot cohort prospective study in a pediatric population with severe burns. We managed to obtain financial support and we succeeded in enrolling and completely processing data from 32 patients in the study group and 20 patients in the control group. We still have been enrolling patients and collecting biological samples, which have been preserved.

The main aim of our study was to evaluate and describe the variations of the CRP, PAI-1, AGP, and PF4 serum levels at three different moments after the burn trauma: 48 h, 10 days, and 21 days. Moreover, these parameters were to be correlated with some clinical parameters (TBSA—total burned surface area, gender, mechanism of burn, length of the hospitalization, the period of time necessary for complete closure of the burn wounds), and with some biochemical parameters such as fibrinogen serum levels, leucocyte count, thrombocyte count, triiodothyronine, thyroxine, thyroid-stimulating hormone serum levels, glomerular filtration rate, prothrombin time, INR, alanine amino-transferase serum levels, and aspartate-amino-transferase serum levels. A statistical analysis was applied upon the results in order to draw conclusions upon (a) the behavior of CRP, PAI-1, AGP, and PF4 in the first 3 weeks post-burn; (b) their predictive value in severe burns in the pediatric population; (c) their correlations with clinical and paraclinical parameters.

## 2. Materials and Methods

### 2.1. Patients Included in This Research

An observational cohort prospective study was performed between 2022 and 2023. Using the following inclusion and exclusion criteria, we enrolled 32 children, from the burn unit of Grigore Alexandrescu Children’s Emergency Hospital in Bucharest. This study was performed after obtaining the Agreement of the Ethics Committee of Grigore Alexandrescu Hospital no. 28656/12.10.2021.

### 2.2. Inclusion and Exclusion Criteria for the Children with a Burn (Study Group)

#### 2.2.1. Inclusion Criteria: Children with a Burn (Study Group)

- Age below 18 years old;
- Thermal burns involving at least 25% TBSA;
- Patients admitted to the burn unit in less than 48 h from the moment of burn infliction;
- The patient’s parents or legal guardian read, understood, and signed the informed consent that states their agreement for the enrolment of their child/children into the present study;
- Patient agreement to be part of this study.

#### 2.2.2. Exclusion Criteria: Children with a Burn (Study Group)

- Pre-existing autoimmune health condition.
- Local or systemic infection at the moment of admission into the burn unit;
- Pre-existing oncologic condition;
- Patients who have been receiving hormonal treatment;
- Patients who have been receiving oncologic treatment;
- Patients who have been receiving immunosuppressive therapy;
- Refusal of the parents or legal guardians to enroll the patient into the present study;
- Refusal of the patient to be included in this study.

### 2.3. Inclusion and Exclusion Criteria for the Control Group

#### 2.3.1. Inclusion Criteria: Control Group

- Age below 18 years old;
- The individual agrees to be included in the control group;
- The patient’s parents or legal guardians read, understood, and signed the informed consent that states their agreement for the enrolment of their child/children into the present study.

#### 2.3.2. Exclusion Criteria: Control Group

- Inflammatory systemic condition.
- Autoimmune health condition;
- Local or systemic infection;
- Oncologic condition;
- Individual under hormonal treatment, or oncologic treatment, or immunosuppressive therapy;
- Individuals with oral health conditions (teeth, gums, mucosa);
- Refusal of the parents or legal guardians to enroll the patient in the present study;
- Refusal of the individual to be part of this study.

After applying the criteria mentioned above, 21 subjects, from the pediatric ward of the same hospital, were included in the control group.

### 2.4. Sample Collection

#### 2.4.1. Sample Collection in the Study Group

For each patient, samples of blood were obtained at three different moments:

- Forty-eight hours after the burn trauma (T1);
- Ten days after the burn trauma (T2);
- Twenty-one days after the burn trauma (T3).

#### 2.4.2. Sample Collection in the Control Group

For the subjects in the control group, samples of blood were also obtained, during the hospitalization in the pediatric ward.

### 2.5. Sample Preservation

The samples were fast-freezed at −20 degrees Celsius in the above-mentioned hospital. Then, the samples were transported on ice, in isotherm bags, and stored at −80 degrees Celsius in the Biochemistry laboratory freezer.

### 2.6. Sample Analyzing and Data Collecting Using Multiplex Technique

The PAI-1, AGP, CRP, and PF4 (among another 12 biochemical parameters, not presented in this paper) were detected using the Multiplex technique. All the samples, standards, and controls were brought to room temperature and incubated with an assay buffer and specific beads overnight at 4 °C with shaking. After removing all the content from the wells and washing the plate three times, the detection antibody was added followed by a 1 h incubation. The streptavidin–phycoerythrin was added with a 30 min incubation. In the end, all the content from the wells was removed, the plate was washed three times again, sheath fluid was added, and the mean florescence intensity (MFI) was read on Luminex 200. All the concentrations are expressed in ng/mL.

Several paraclinical parameters were selected from the pool of paraclinical investigations performed for each patient: fibrinogen, leucocyte count, thrombocyte count, triiodothyronine, thyroxine, thyroid-stimulating hormone, prothrombin time, INR, aspartate aminotransferase (AST), alanine aminotransferase (ALT), blood urea, and blood creatinine.

We collected several clinical and epidemiological types of data, which we retained for our study: TBSA, hospitalization period length, age, and burn mechanism.

All the data collected from the children’s samples in the study group and from the subjects’ samples in the control group were anonymized.

### 2.7. Data Analysis

All the data from this study were analyzed using IBM SPSS Statistics 25 and illustrated using Microsoft Office Excel/Word 2021. Quantitative variables were tested for normal distribution using the Shapiro–Wilk test and were written as averages with standard deviations or medians with interquartile ranges. Quantitative variables were tested between measurements using Friedman’s tests along with Dunn–Bonferroni post hoc tests. Quantitative independent variables were tested between groups using Mann–Whitney U tests, and correlations between them were calculated using the Spearman’s rho correlation coefficient. Qualitative variables were written as absolute frequencies with percentages and were tested between groups using Fisher’s Exact Test.

## 3. Results

Data from Table 1 show the characteristics of the studied groups. A total of 32 patients (60.4%) were included in the study group and 21 patients (39.6%) were included in the control group. Differences of gender frequencies between groups were not statistically significant according to the Fisher test (*p* = 0.089) while patients in the study group were significantly younger (median = 3 years, IQR = 2–10) in comparison to patients in the control group (median = 14 years, IQR = 12–16) according to the Mann–Whitney U test (*p* < 0.001).

In the study group, the median hospitalization period was 35 days (interquartile range = 25–56 days) and the median burned body surface percentage was 35% (interquartile range = 27–45%) while the median time from the event to hospitalization was 8 h (interquartile range = 4–9.5 h). The distribution according to the burn injury mechanism shows that the majority of patients suffered burns caused by hot liquids (46.9%) or flames (40.6%).

**Figure 1:**
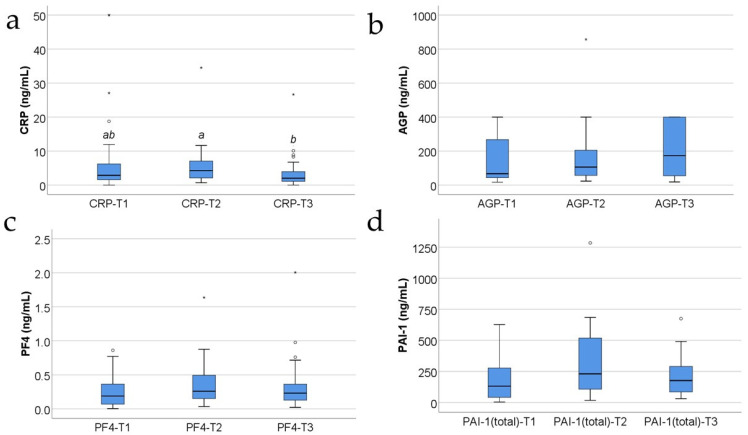
The box-plot illustration for CRP (**a**), AGP (**b**), PF4 (**c**), and PAI-1 (**d**) evolutions in the study group (concentrations expressed in ng/mL). Values are medians with interquartile ranges. The statistical analysis by Friedman’s tests with Dunn–Bonferroni post-tests. Different lower-case letters indicate a significant difference between measurements. There were no significant differences between measurements in the case of AGP (*p* = 0.466), PF4 (*p* = 0.381), or PAI-1 (*p* = 0.055). In the box-plot figure, an outlier is represented with the ° symbol when the value is higher than the 3rd quartile + 1.5IQR or lower than the 1st quartile − 1,5IQR. An extreme outlier is represented with the * symbol when the value is higher than the 3rd quartile + 3IQR or lower than the 1st quartile − 1.5IQR.

Figure 1 shows the evolution of analyzed inflammatory parameters in the study group. The distribution of the analyzed variables was non-parametric according to the Shapiro–Wilk test across most of the measurements (*p* < 0.05). Differences between measurements were significant only for CRP measurements according to the Friedman test (*p* = 0.014), and post hoc tests show that values measured at T3 (median = 2, IQR = 0.9–4.1) were significantly lower than those measured at T2 (median = 4.2, IQR = 1.7–7.2) (*p* = 0.012) but differences between T1 and T2 (*p* = 0.881) or T1 and T3 (*p* = 0.198) were not significant. The evolution of AGP (*p* = 0.466) and PF4 (*p* = 0.381) measurements was not significant while there was an observed tendency towards statistical significance for PAI-1 measurements (*p* = 0.055) in the direction of a higher value at T2 versus T1, but the limited number of patients could not demonstrate the significance.

**Figure 2:**
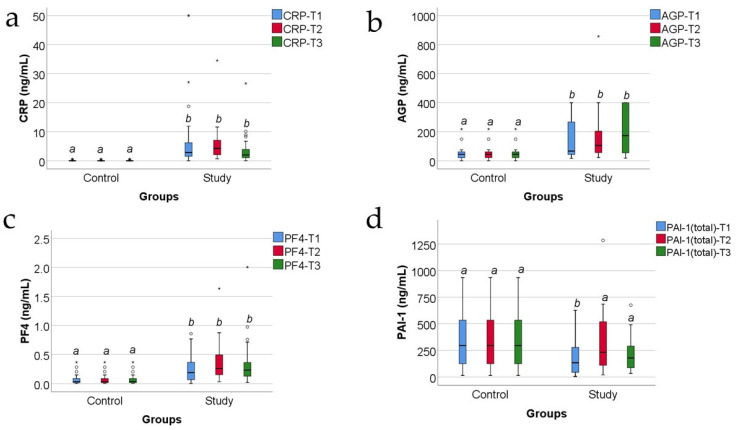
The comparison of CRP (**a**), AGP (**b**), PF4 (**c**), and PAI-1 (**d**) measurements between control and study groups (concentrations expressed in ng/mL). Values are medians with interquartile ranges. The statistical analysis by Mann–Whitney U tests. Different lower-case letters indicate a significant difference between groups. In the box-plot figure, an outlier is represented with the ° symbol when the value is higher than the 3rd quartile + 1.5IQR or lower than the 1st quartile – 1,5IQR. An extreme outlier is represented with the * symbol when the value is higher than the 3rd quartile + 3IQR or lower than the 1st quartile – 1.5IQR.

Figure 2 shows the comparison of analyzed inflammatory parameters between the study and control group. The distribution of the analyzed variables was non-parametric according to the Shapiro–Wilk test across most of the measurements in both groups (*p* < 0.05). Differences between groups were significant for all parameters, showing higher values of CRP (*p* < 0.001/*p* < 0.001/*p* < 0.001), AGP (*p* = 0.002/*p* < 0.001/*p* = 0.001), and PF4 (*p* = 0.001/*p* < 0.001/*p* < 0.001) at all measurements in the study group versus the control group and lower values of PAI-1 at T1 (*p* = 0.015) in the study group versus the control group.

**Figure 3:**
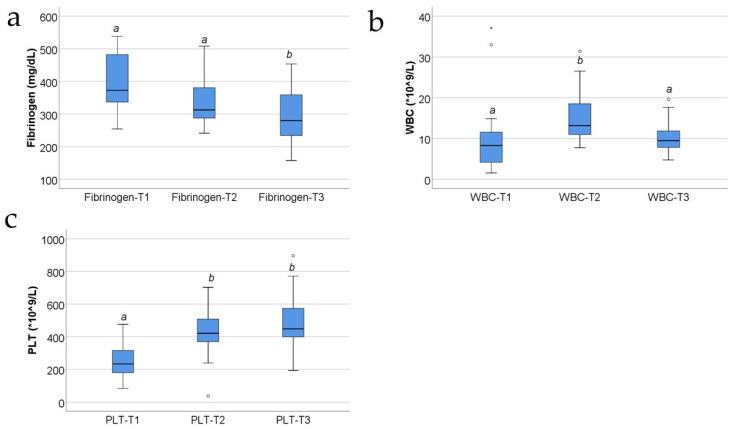
The box-plot illustration for fibrinogen (**a**), WBC (**b**), and platelet (**c**) evolutions in the study group (concentrations expressed in mg/dL (**a**) and 10^9^ cells/L (**b**,**c**)). Values are medians with interquartile ranges. The statistical analysis by Friedman’s tests with Dunn–Bonferroni post-tests. Different lower-case letters indicate a significant difference between measurements. In the box-plot figure, an outlier is represented with the ° symbol when the value is higher than the 3rd quartile + 1.5IQR or lower than the 1st quartile – 1,5IQR. An extreme outlier is represented with the * symbol when the value is higher than the 3rd quartile + 3IQR or lower than the 1st quartile – 1.5IQR.

**Figure 4:**
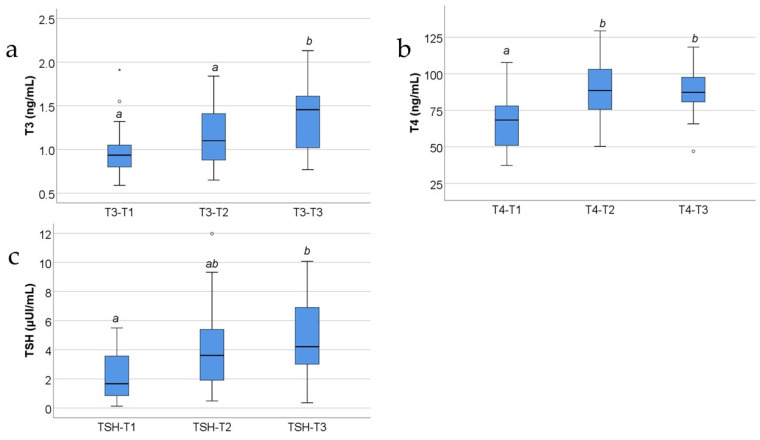
The box-plot illustration for triiodothyronine (**a**), thyroxine (**b**), and TSH (**c**) evolutions in the study group (concentrations expressed in ng/mL (**a**,**b**) and μUI/mL (**c**)). Values are medians with interquartile ranges. The statistical analysis by Friedman’s tests with Dunn–Bonferroni post-tests. Different lower-case letters indicate a significant difference between measurements. In the box-plot figure, an outlier is represented with the ° symbol when the value is higher than the 3rd quartile + 1.5IQR or lower than the 1st quartile – 1,5IQR. An extreme outlier is represented with the * symbol when the value is higher than the 3rd quartile + 3IQR or lower than the 1st quartile – 1.5IQR.

Figure 3 and Figure 4 show the evolution of usual serum parameters in the study group. The distribution of the analyzed variables was non-parametric according to the Shapiro–Wilk test across most of the measurements (*p* < 0.05). Differences between measurements were significant for all parameters, according to the Friedman tests, and the data from post hoc tests show the following:

- Fibrinogen and triiodothyronine concentrations were significantly lower at T3 than at T2 (*p* = 0.015/*p* = 0.025) or T1 (*p* < 0.001/*p* = 0.004);
- White blood cells (WBCs) were significantly increased at T2 compared to at T1 (*p* < 0.001) or T3 (*p* = 0.007);
- Platelets (PLTs) and thyroxine were significantly increased at T2 (*p* < 0.001/*p* < 0.001) or T3 (*p* < 0.001/*p* < 0.001) compared to at T1;
- TSH values were significantly higher at T3 compared to at T1 (*p* = 0.004).

**Figure 5:**
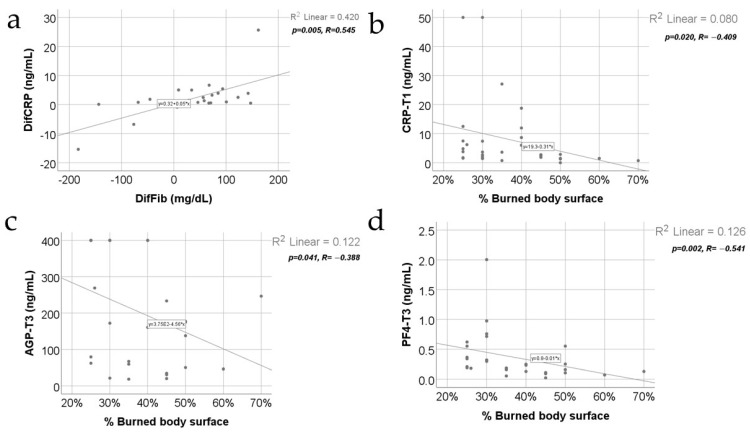
Correlation between CRP and fibrinogen evolution from T2 to T3 (**a**) along with correlations between percentage of burned body surface and CRP value at T1 (**b**), AGP value at T3 (**c**), and PF4 value at T3 (**d**).

Figure 5 shows the correlations between CRP evolution from T2 to T3 and fibrinogen, WBC, and triiodothyronine evolutions from T2 to T3. The distribution of the CRP difference was non-parametric according to the Shapiro–Wilk test (*p* < 0.001). The observed correlations were not significant between CRP evolution and WBC evolution (*p* = 0.336) or CRP evolution and triiodothyronine evolution (*p* = 0.633), but in the case of CRP and fibrinogen evolutions, the observed correlation was significant and positive, having a moderate power (*p* = 0.005, R = 0.545), indicating that patients who had higher differences of CRP from T2 to T3 were significantly more associated with higher differences of fibrinogen from T2 to T3 and vice versa.

Data from Figure 5 also show the correlations between inflammatory parameters and the percentage of the burned body surface. The distribution of the burned body surface percentage was non-parametric according to the Shapiro–Wilk test (*p* = 0.003). Most of the observed correlations were not statistically significant, except for the following significant and negative correlations between the percentage of the burned body surface and CRP value at T1 (*p* = 0.020, R = −0.409), AGP value at T3 (*p* = 0.041, R = −0.388), and PF4 value at T3 (*p* = 0.002, R = −0.541), which show that patients with high percentages of the burned body surface were significantly more associated with low values for CRP at T1/AGP at T3/PF4 at T3 while patients with low percentages of the burned body surface were significantly more associated with high values for CRP at T1/AGP at T3/PF4 at T3.

**Figure 6:**
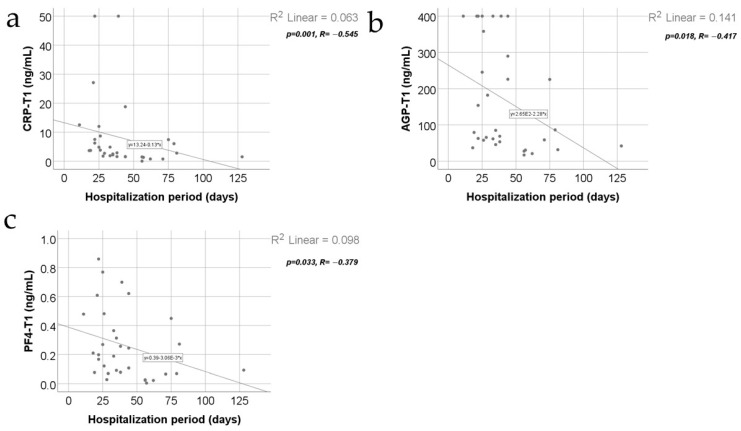
Correlations between hospitalization period and CRP value at T1 (**a**), AGP value at T1 (**b**), and PF4 value at T1 (**c**).

Figure 6 shows the correlations between inflammatory parameters and hospitalization period. The distribution of the hospitalization period was non-parametric according to the Shapiro–Wilk test (*p* = 0.001). Most of the observed correlations were not statistically significant, except for the following significant and negative correlations between the hospitalization period and CRP value at T1 (*p* = 0.001, R= −0.545), AGP value at T1 (*p* = 0.018, R= −0.417), and PF4 value at T1 (*p* = 0.033, R= −0.379), which show that patients with longer hospitalization were significantly more associated with low values for CRP/AGP/PF4 at T1 while patients with shorter hospitalization were significantly more associated with high values for CRP/AGP/PF4 at T1.

**Figure 7:**
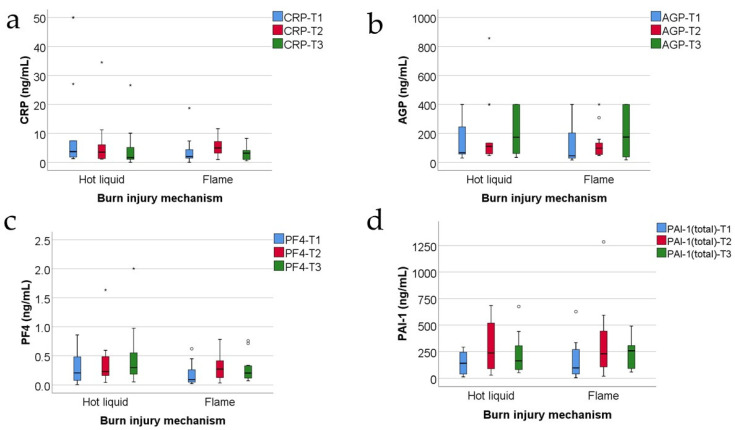
The comparison of CRP (**a**), AGP (**b**), PF4 (**c**), and PAI-1 (**d**) measurements between patients according to the burn injury mechanism (concentrations expressed in ng/mL). Values are medians with interquartile ranges. The statistical analysis by Mann–Whitney U tests. There were no observed significant differences between groups (*p* > 0.05). In the box-plot figure, an outlier is represented with the ° symbol when the value is higher than the 3rd quartile + 1.5IQR or lower than the 1st quartile − 1.5IQR. An extreme outlier is represented with the * symbol when the value is higher than the 3rd quartile + 3IQR or lower than the 1st quartile − 1.5IQR.

Figure 7 shows the comparison of inflammatory parameters between patients according to the burn injury mechanism. The distribution of the analyzed variables was non-parametric according to the Shapiro–Wilk test across most of the measurements in both groups (*p* < 0.05). Differences between groups were not significant for all parameters according to the Mann–Whitney U tests (*p* > 0.05); as such, none of the parameters at any of the measurements were significantly different between patients with burn injuries caused by hot liquids or flames.

**Figure 8:**
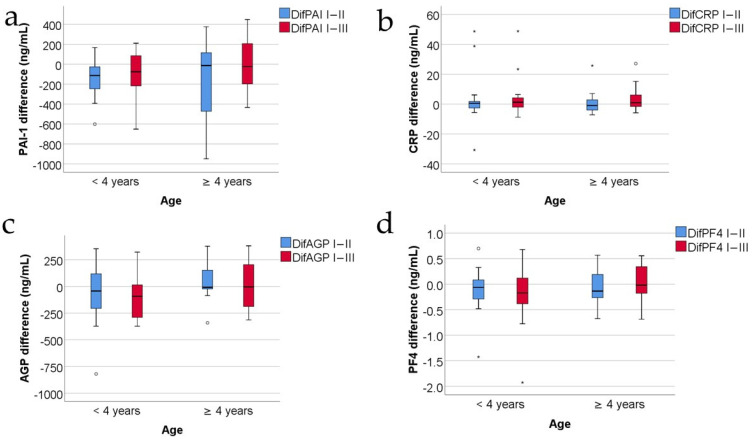
The comparison of CRP (**a**), AGP (**b**), PF4 (**c**), and PAI-1 (**d**) evolutions between patients according to age (concentrations expressed in ng/mL). Values are medians with interquartile ranges. The statistical analysis by Mann–Whitney U tests. No observed significant differences between groups (*p* > 0.05). In the box-plot figure, an outlier is represented with the ° symbol when the value is higher than the 3rd quartile + 1.5IQR or lower than the 1st quartile − 1,5IQR. An extreme outlier is represented with the * symbol when the value is higher than the 3rd quartile + 3IQR or lower than the 1st quartile − 1.5IQR.

Figure 8 shows the comparison of analyzed parameter evolutions between patients according to age: ages below 4 years versus ages above 4 years. According to the results, none of the parameters were significantly different between the age groups (*p* > 0.05).

**Figure 9:**
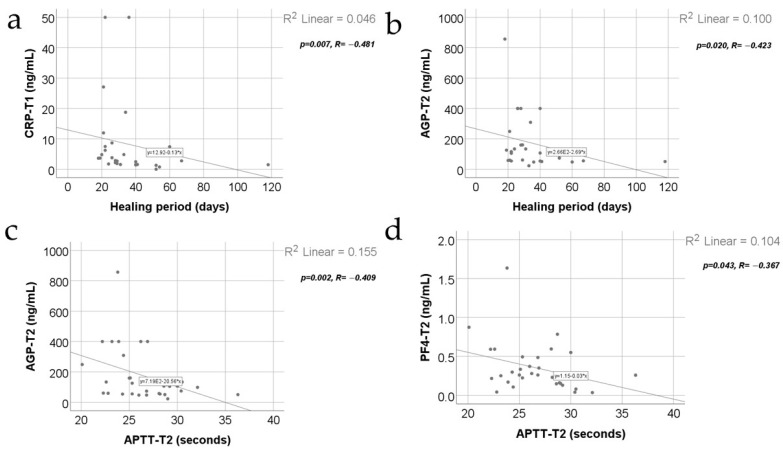
Correlations between the healing period and CRP value at T1 (**a**) and AGP value at T2 (**b**) along with the correlations between the APTT value at T2 and AGP value at T2 (**c**) and PF4 value at T2 (**d**).

Figure 9 shows the correlations between inflammatory parameters and the healing period along with the APTT value at T2. The distribution of the period was non-parametric according to the Shapiro–Wilk test (*p* < 0.001). Most of the observed correlations were not statistically significant, except for the significant and negative correlations between the CRP measurement at T1 and the healing period (*p* = 0.007, R = −0.481) or the AGP measurement at T2 and the healing period (*p* = 0.020, R = −0.423), which shows that patients with high values of CRP at T1 or AGP at T2 were significantly more associated with low values for the healing period and vice versa. Also, significative negative correlations were observed between the APTT value at T2 and AGP value at T2 (*p* = 0.002, R = −0.409) or APTT value at T2 and PF4 value at T2 (*p* = 0.043, R = −0.367), which shows that patients with high values of APTT were significantly more associated with lower values of AGP and PF4 and vice versa.

**Figure 10:**
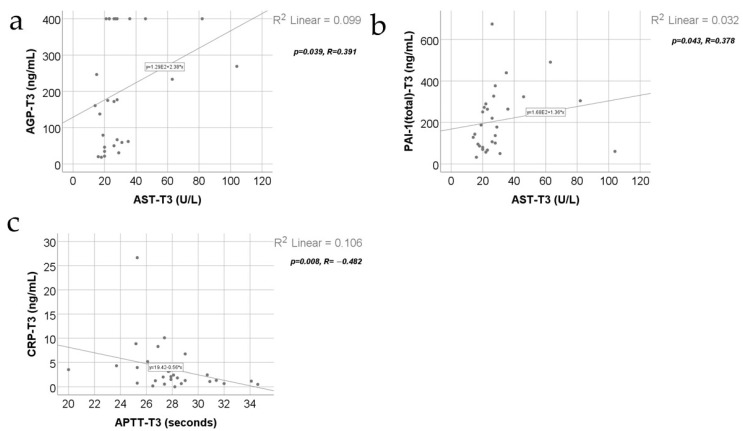
Correlations between the AST value at T3 and AGP value at T3 (**a**) and PAI-1 value at T3 (**b**) along with the correlation between the APTT value at T3 and CRP value at T3 (**c**).

Figure 10 shows the correlations between inflammatory parameters and the AST value at T3 along with the APTT value at T3. Between the AST value at T3 and AGP value at T3 (*p* = 0.039, R = 0.391) or AST value at T3 and PAI-1 value at T3 (*p* = 0.043, R = 0.378), positive significant correlations were observed, which shows that patients with high values of AST were significantly more associated with higher values of AGP or PAI-1 and vice versa. Also, between the APTT value at T3 and CRP value at T3 (*p* = 0.008, R = −0.482), a negative significant correlation was observed, which shows that patients with high values of APTT were significantly more associated with lower values of CRP and vice versa.

## 4. Discussion

Besides studying the evolution of the serum levels of the four molecules (CRP, AGP, PAI-1, and PF4) of interest for our research, and comparing the results of the study group with those of the control group, we also correlated these data with other parameters, such as TBSA, gender, mechanism of burn, length of the hospitalization, serum levels of fibrinogen, triiodothyronine, thyroxine, TSH, and WBC and PLT count, at 48 h (T1), 10 days (T2), and 21 days (T3) post-burn. WBC, PLT, and fibrinogen serum levels have been commonly used to follow the evolution of the systemic inflammatory response in severe burns, in other types of severe trauma, and in sepsis. We also searched for correlations of CRP, PAI-1, AGP, and PF4 with (a) the hepatic function (evaluated through AST and ALT serum levels, prothrombin time (PT), Activated Partial Thromboplastin Time (APTT), and International Normalized Ratio (INR) blood test); (b) the kidney function (evaluated through Estimated Glomerular Filtration Rate (GFR)).

Regarding triiodothyronine, thyroxine, and TSH levels, in severe burns, two possible situations are described: (a) In the first situation, which is the most common, triiodothyronine and thyroxine levels significantly decrease in the first two weeks, accompanied by a significant increase in TSH. So, severe burn trauma induces a hypothyroidism-like state in the first 14–21 days post-burn. Only after the third week do the triiodothyronine and thyroxine levels slowly increase to normal values and TSH levels slowly decrease towards normal values [1,39,40,41]. The persistency of the low serum levels of the thyroid hormones, after day 21 post-burn, is linked to a bad prognosis [1,39,40,41]. (b) In the second situation, which is rarer, the severe burn induces an acute thyrotoxicosis crisis called a “thyroid storm” that accentuates the systemic disturbances and could kill the patient [42,43]. This happens when the patient already has a hyperthyroidism that is exacerbated by the burn trauma. That is why we wanted to describe the alterations of the serum levels of triiodothyronine, thyroxine, and TSH and to investigate if there were any correlations with the variations of CRP, AGP, PAI-1, and PF4 serum levels in the study group. In our study, we noticed that triiodothyronine serum levels constantly increase at 48 h, at 10 days, and further at 21 days (Figure 4a). Thyroxine serum levels increase at 48 h and at 10 days and then have a slow decrease towards 21 days (Figure 4b). TSH serum levels, just like triiodothyronine levels, constantly increase at 48 h, at 10 days, and further at 21 days (Figure 4c). The results of our research contradict previous studies that showed a dramatic decrease in triiodothyronine and thyroxine serum levels in the first 14–21 days after the burn trauma, followed by an increase after day 21. Concerning TSH serum levels, our results confirm previous studies, showing a steady increase in the first three weeks post-burn.

CRP is one of the most studied acute-phase proteins in severe trauma, including burns [10,44,45,46,47]. It is considered to be a marker of the inflammatory response of the patient, not specifically related to sepsis [8,47]. In our study, the serum CRP levels increased at T1 and further increased from T1 to T2, and then decreased towards T3, following the good evolution and the decrease in the inflammatory response (Figure 1a and Figure 2a). As expected, the CRP levels were higher in the study group, versus the control group (Figure 2a).

The evolution of CRP serum levels resembles that of the WBC count and thyroxine serum levels in the study group, showing an increase at T1, then a further increase at T2 compared to T1 and then a decrease at T3 compared to T2 (Figure 1a compared with Figure 3b, Figure 1 compared with Figure 4b). But the thyroxine levels at T3 remain higher than those measured at T1, unlike the CRP levels at T3 that decrease almost to the levels measured at T1.

The evolution of CRP serum levels, which demonstrates an increase at T1, with a further increase at T2 compared with T1, and then a decrease at T3 compared to T2 (Figure 1a), does not follow the evolution of the serum levels of triiodothyronine, the serum levels of TSH, and the platelet count, which constantly increase at T2 compared to T1 and at T3 compared to T2 (Figure 4a, Figure 4c and Figure 3c, respectively).

The evolution of serum CRP levels does not resemble the evolution of fibrinogen levels in the study group, the latter presenting a tendency to decrease, T1 > T2 > T3 (Figure 1a compared with Figure 3a).

In our study, there was a negative correlation between TBSA and CRP serum levels at T1: patients with lower TBSA were significantly associated with high values for CRP at 48 h post-burn (Figure 5b). Our results also demonstrated a negative correlation between the hospitalization length and CRP serum levels at T1: patients with a shorter hospitalization period were significantly more associated with high values for CRP at 48 h post-burn (Figure 6a).

In our study (Figure 9a), there was a statistically significant negative correlation between CRP values at T1 and the healing period (defined as the period of time measured from the moment of burn infliction to the burn wounds being completely covered by re-epithelization, scarring, or skin grafting). The correlation that we found means that the higher the serum CRP levels at 48 h post-burn, the shorter the healing period (the faster the healing process).

The evolution of serum CRP levels in the study group is different related to the burn mechanism: a lower level at T2 compared to T1 and lower level at T3 compared to T2 for injuries produced with hot liquid and higher level at T2 compared to T1 and lower level at T3 compared to T2 for burns produced by flame (Figure 7a), but these differences were not statistically significant.

CRP serum levels at T3 and APTT values at T3 presented a statistically significant and negative correlation (Figure 10c). This means that patients with higher CRP serum levels at 21 days had shorter APTT. In other words, a higher CRP at 21 days post-burn predicted a tendency toward hypercoagulability, reflected in a shorter APTT.

We did not find any statistically significant correlations between CRP serum levels and GFR, AST, ALT, PT, and INR.

Our results do not correspond with the results of Jeschke and colab. from other studies on CRP in pediatric burns [8]: we found that higher CRP levels negatively correlate with burn size, but we could not confirm higher CRP levels’ correlation with non-survivors and with female gender, as the number of patients enrolled in this pilot study is smaller and the follow-up was not long enough (3 weeks versus 6 months in other studies). We found that, indeed, CRP rises immediately after burn trauma, but as our study followed the levels only until 21 days post-burn, we could not confirm other studies’ results that showed increased levels of CRP up to 270 days after the burn [48]. An observation of ours that we consider interesting is that the evolution of CRP levels is different between the subgroup of burns through hot liquids and the subgroup of burns through flame. We could not correlate our observation with other studies.

PAI-1 is an acute-phase protein. It is a serine protease inhibitor that acts as a fibrinolytic inhibitor. In severe burns, two apparently opposed tendencies are noted: on one hand, there is a rapid increase in pro-fibrinolytic activity and on the other hand, one can note the increase in anti-fibrinolytic factor levels [14]. Basically, PAI-1 has a procoagulant effect [49]. In the first 48 h after the burn, an increase in PAI-1 levels was reported, followed by a steady decrease after 48 h, in the case of favorable evolution [50].

In our study, PAI-1 increases from T1 to T2, and only after 10 days post-burn starts to decrease, so the values at T3 are smaller compared with T2 values (Figure 1d). A totally unexpected finding is that the PAI-1 levels were lower in the study group versus control group (Figure 2d), except for T2 when the serum levels were almost similar.

The evolution of PAI-1 serum levels resembles that of the WBC count and thyroxine serum levels in the study group, showing an increase at T2 compared to T1 and then a decrease at T3 compared to T2 (Figure 1d compared with Figure 3b, Figure 1d compared with Figure 4b).

The evolution of PAI-1 serum levels, which demonstrates an increase at T2 compared with T1 and a decrease at T3 compared to T2 (Figure 1d), does not follow the evolution of the serum levels of triiodothyronine, the serum levels of TSH, and the platelet count, which constantly increase at T2 compared to T1 and at T3 compared to T2 (Figure 1d compared to Figure 4a, Figure 1d compared to Figure 4c, and Figure 1d compared to Figure 3c, respectively).

The evolution of serum PAI-1 levels does not resemble the evolution of fibrinogen levels in the study group, the latter presenting a tendency to decrease, T1 > T2 > T3 (Figure 1d compared with Figure 3a). The progression of serum PAI-1 levels in the study group is not different related to the burn mechanism: a lower level at T2 compared to T1 and lower level at T3 compared to T2, both in the subgroup with hot liquid burns and in the subgroup with flame burns (Figure 7d). Moreover, higher levels of serum PAI-1 were not necessarily observed in patients with larger burns.

In our study, we found that PAI-1 levels increase immediately after the burn, but start decreasing only after day 10 post-burn, unlike other studies that reported, in patients with favorable evolution, a continuous decrease in PAI-1 serum levels after 48 h [49,50], or after 5 days [51]. Being the main inhibitor of plasminogen activators, PAI-1 plays a central role in the regulation of the fibrinolytic system [52]. Its increased levels until day 10 post -burn explain, among other factors, the procoagulant state of the patients with a severe burn that has been described and documented in many articles and monographs [14,53]. In our study, we could not find any statistically significant correlation between the PAI-1 serum levels and TBSA. Also, we could not prove a statistically significant correlation between the length of hospitalization or the healing period and PAI-1 serum levels.

We found a statistically significant and positive correlation between PAI-1 serum levels at T3 and AST (Figure 10b), meaning that the higher the PAI-1 serum levels at 21 days, the greater the probability for the patient to have an increased AST serum level. An increased AST serum level (usually associated with other biochemical changes) reflects a degree of liver insufficiency.

We did not find any statistically significant correlation between PAI-1 and the estimated GFR.

AGP is an acute-phase protein. Its serum levels increase in severe trauma, including burns, surgical stress, infection, inflammation, and malignancies [23,24,25,26]. AGP was proposed as a biomarker in the prediction of all-cause mortality [27,28]. It is also an important protein that binds different molecules, including drugs, and it exerts an immunomodulatory effect. Furthermore, AGP has an inhibitory action upon platelet aggregation [27]. Some studies demonstrated an anti-inflammatory effect of AGP [29].

In our study, AGP increases at T1, then decreases from T1 to T2, and after 10 days post-burn starts to increase, so the values at T3 are higher compared with T2 and compared with T1 values (Figure 1b). Thus, the evolution of the serum levels of AGP in our study shows an initial raise in the first 48 h, followed by a decrease by the 10th day, and then a constant rise towards the end of the third week after the burn trauma. It is unclear why, despite the good evolution of the patients, the AGP serum levels start rising again after day 10. One might speculate that the serial surgical “excision and grafting” or “degranulation and grafting” procedures might be responsible for this otherwise unexplainable rise in AGP serum levels after day 10; in the study group, the majority of the patients benefited from surgical procedures around the 8th day post-burn and/or between the 10th and the 21st day post-burn. As expected, the AGP levels were increased in the study group versus control group (Figure 2b).

The evolution of AGP serum levels (Figure 1b) is different when compared with the variation of the serum levels of fibrinogen, which are increased at T1, then constantly decrease at T2, and further decrease at T3 (Figure 3a). Additionally, it does not resemble the evolution of the WBC count, which increases at T2 compared to T1, then decreases at T3 compared to T2 (Figure 3b), nor thyroxine serum levels in the study group, which show an increase at T2 compared to T1 and then a decrease at T3 compared to T2 (Figure 4b).

The evolution of AGP serum levels does not follow the evolution of the serum levels of triiodothyronine, the serum levels of TSH, and the platelet count either, which constantly increase at T2 compared to T1 and at T3 compared to T2 (Figure 4a, Figure 4c and Figure 3c, respectively). Moreover, in our study, the AGP serum levels in the study group do not positively correlate with the TBSA percentage involved in the burn (Figure 5c).

There are no differences in the variation pattern of the serum levels of AGP in the subgroup with hot liquid burns when compared with the subgroup with flame burns (Figure 7b). The serum AGP levels do not positively correlate with the length of hospitalization (Figure 6b). In fact, the hospitalization period negatively correlates with the AGP levels at T1. This means that the higher the AGP levels at 48 h post-burn, the shorter the hospitalization.

Regarding the AGP serum levels’ correlation with TBSA (as one of the main determinants, together with the burn depth, of the severity of burns), two situations are reported: (a) the first situation, in which the intensity of the acute-phase response, including the AGP synthesis, increases with the severity of the burn [54]; (b) the second situation, in which the AGP serum levels do not correlate with the TBSA [55]. In our study, TBSA negatively correlates with the AGP serum levels at T3, meaning that patients with lower TBSA were significantly more associated with high values for AGP at 21 days post-burn (Figure 5c).

We found a statistically significant and negative correlation between the AGP serum levels at T2 and the healing period (Figure 9b), meaning that high values of AGP at 10 days were significantly more associated with a shorter healing period. In other words, the higher the AGP serum levels at 10 days post-burn, the faster the healing process.

The inhibitory effect of high AGP concentrations upon TSH signaling, by interacting directly with the TSH receptors at the level of the thyroid cell, was reported by previous studies [56,57]. This affirmation might be sustained by our study that shows significant higher levels of serum AGP and serum TSH at T3 versus T1 and T2. The higher TSH levels reflected correspondent higher thyroxine and triiodothyronine levels at 21 days post-burn.

We found a statistically significant and negative correlation between the AGP serum levels at T2 and APTT at T2 (Figure 9c), meaning that the higher the AGP serum levels at 10 days, the lower the APTT values. In other words, an increased AGP at 10 days post-burn associates with an increased risk of hypercoagulability, reflected by a decreased APTT.

We found a statistically significant and positive correlation between AGP serum levels at T3 and AST (Figure 10a), meaning that the higher the AGP serum levels at 21 days, the greater the probability for the patient to have an increased AST serum level. An increased AST serum level (usually associated with other biochemical changes) reflects a degree of liver insufficiency.

We did not find any statistically significant correlation between AGP and the estimated GFR.

Platelet factor 4 (PF4) is an acute-phase protein, a small cytokine belonging to the CXC chemokine family that is also known as chemokine (C-X-C motif) ligand 4 (CXCL4) [34,35]. It promotes blood coagulation, and it also has immunomodulatory effects [34,36,58]. It appears that, in the first day after severe trauma, there is an upregulation of the gene encoding the synthesis of PF4 in dendritic cells [34].

In our study, the serum PF4 levels increased at T1 and further increased from T1 to T2, and then decreased towards T3, following the good evolution and the decrease in the inflammatory response (Figure 1c). As expected, the PF4 levels were higher in the study group, versus the control group (Figure 2c).

The evolution of PF4 serum levels resembles that of the WBC count and thyroxine serum levels in the study group, showing an increase at T1, then a further increase at T2 compared to T1 and then a decrease at T3 compared to T2 (Figure 1c compared with Figure 3b, Figure 1c compared with Figure 4b). But the thyroxine levels at T3 remain higher than thyroxine levels at T1, unlike the PF4 levels at T3 that drop almost to the levels measured at T1.

The evolution of PF4 serum levels does not follow the evolution of the serum levels of triiodothyronine, the serum levels of TSH, and the platelet count, which constantly increase at T2 compared to T1 and at T3 compared to T2 (Figure 4a, Figure 4c and Figure 3c, respectively), in situations of favorable evolution.

Related to the burns’ mechanism, PF4 levels in the subgroup of burns produced by hot liquid were higher than PF4 levels in the subgroup of burns produced by flame (Figure 7c). Additionally, the PF4 levels negatively correlate with the length of the hospitalization period at T1 (Figure 6c); thus, patients with higher PF4 levels at 48 h post-burn had a shorter period of hospitalization (Figure 6c).

We did not find any statistically significant correlation between PF4 serum levels and the length of the healing period.

The procoagulant effect of PF4, which appears to be immune-mediated, was demonstrated by several studies [59,60]. Interestingly, this small chemokine was proven to have both pro-inflammatory effects [60,61] and anti-inflammatory effects [62]. In our study, it appears that PF4 levels increase after day 10, seeming to be a hallmark of a favorable evolution. This observation might be related to the anti-inflammatory action of FP4 communicated by several authors [63]. Such a conclusion (PF4 heralding a favorable evolution) pleads for the negative correlation of the length of hospitalization (Figure 6c) with the serum levels of PF4 and, also, the negative correlation of TBSA with the serum levels of PF4 (Figure 5d): patients with low TBSA were significantly more associated with high values of PF4 at 21 days post-burn (Figure 5d).

We found a statistically significant and negative correlation between the PF4 serum levels at T2 and APTT at T2 (Figure 9d), meaning that the higher the PF4 serum levels at 10 days, the lower the APTT values. In other words, an increased PF4 serum level at 10 days post-burn associates with an increased risk of hypercoagulability, reflected by a decreased APTT.

Several studies proved that children under 4 years of age have a higher mortality than children older than 4 years [64]. On the other hand, Jeschke and colab. reported in 2013 that children under 4 years of age exhibit higher CRP [8] and Song et al. reported higher CRP levels and higher mortality in children and in the elderly [9]. That is why we verified if there were any significant differences between the subgroup of children below 4 years of age and the subgroup of children at a minimum of 4 years of age, concerning the levels and evolution of the CRP, AGP, PAI-1, and PF4. We found that neither CRP nor AGP, PAI-1, or PF4 exhibited any differences between the two subgroups of children with a burn: below and above the age of 4 years (Figure 8a–d).

The main limitations of this study are

1. The fact that it was not a multi-center study.
2. The limited number of patients enrolled in this study because this study was conducted during the COVID-19 pandemic and immediately after the pandemic.
3. The patients were studied only during the first 21 days post-burn as the post-discharge follow-up was very difficult to conduct: many patients would not come for scheduled check-ups.
4. The differences of median age of the study group and of the control group.

Therefore, our future perspectives focus on conducting a multi-center study, including pediatric patients and adult patients. Moreover, further studies should enroll a larger number of patients and a longer period of follow-up should be taken into consideration.

## 5. Conclusions

CRP, PAI-1, AGP, and PFP have different roles. This is reflected in the fact that, although all the studied molecules are acute-phase proteins, their serum levels present different curves of variation at 48 h (T1), 10 days (T2), and 21 days (T3). Each of them shows particular and non-identical aspects in the biochemical profile of a patient with a severe burn.

Our study showed statistically significant correlations between CRP, AGP, and PF4 serum levels and the length of hospitalization and TBSA. We did not find any correlation with statistical significance between PAI-1 serum levels and the length of hospitalization nor between PAI-1 serum levels and TBSA.

None of the parameters at any of the measurements were significantly different between patients with burn injuries caused by hot liquids or flames.

We noticed that CRP and AGP serum levels significantly correlated with TBSA and were good predictors for the length of hospitalization and for the length of the healing period, showing that (a) higher CRP values at 48 h post-burn predict faster healing and shorter hospitalization, (b) higher AGP levels at 48 h predict shorter hospitalization, and (c) higher AGP serum levels at 10 days post-burn predict a shorter healing period.

PF4 serum levels significantly and negatively correlated with the length of hospitalization, but not with the length of the healing period.

None of the investigated molecules significantly correlated with the renal function, quantified by the estimated GFR.

Interestingly, AGP and PAI-1 serum levels at 21 days significantly correlated with AST levels (which reflects the hepatic function).

AGP and PF4 serum levels at 10 days significantly and negatively correlated with APTT values, showing a tendency towards hypercoagulability.

Given the facts enumerated above, we consider that CRP, PAI-1, AGP, and PF4 are interesting molecules that help us complete the “puzzle” of the biochemical milieu of a patient with a severe burn. We consider that further studies on larger groups of patients need to be performed, in order to better understand the potential roles of CRP, PAI-1, AGP, and PF4 as serologic markers in severe burns.

## Figures and Tables

**Table 1:** Characteristics of the studied groups.

Group/Parameter	Study Group	Control Group	*p*
Number of patients (Nr., %)	32 (60.4%)	21 (39.6%)	-
Gender—male (Nr., %)	22 (68.8%)	9 (42.9%)	0.089 *
Age (Median (IQR))	3 (2–10)	14 (12–16)	<0.001 **
Hospitalization period (Median (IQR))	35 (25–56)	-	-
% Burned body surface (Median (IQR))	35 (27–45)	-	-
Time from event to hospitalization (Median (IQR))	8 (4–9.5)	-	-
*Burn injury mechanism (Nr., %)*			
Hot liquid	15 (46.9%)	-	-
Flame	13 (40.6%)	-	-
Electric arc	4 (12.5%)	-	-

* Fisher’s Exact Test, ** Mann–Whitney U Test.

## Data Availability

Data supporting the reported results are available from the authors.
